# *N*′-[4-(Dimethyl­amino)benzyl­idene]-3-hydroxy­benzohydrazide

**DOI:** 10.1107/S160053680800130X

**Published:** 2008-01-18

**Authors:** Yi Nie

**Affiliations:** a Department of Chemistry, Qufu Normal University, Qufu 273165, People’s Republic of China

## Abstract

The title compound, C_16_H_17_N_3_O_2_, was synthesized by the reaction of 4-dimethyl­amino­benzaldehyde with 3-hydroxy­benzoic acid hydrazide in methanol. The dihedral angle between the two benzene rings in the mol­ecule is 9.2 (2)°. In the crystal structure, mol­ecules are linked through inter­molecular O—H⋯O, O—H⋯N and N—H⋯O hydrogen bonds, forming layers parallel to the *bc* plane.

## Related literature

For related literature, see: Akitsu & Einaga (2006 ▶); Bahner *et al.* (1968 ▶); Butcher *et al.* (2005 ▶); Hodnett & Mooney (1970 ▶); Merchant & Chothia (1970 ▶); Pradeep (2005 ▶); Sigman & Jacobsen (1998 ▶).
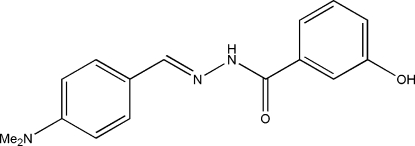


## Experimental

### 

#### Crystal data

C_16_H_17_N_3_O_2_
*M* *_r_* = 283.33
Monoclinic, 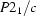

*a* = 13.397 (3) Å
*b* = 9.663 (2) Å
*c* = 11.183 (2) Å
β = 101.97 (3)°
*V* = 1416.2 (5) Å^3^
*Z* = 4
Mo *K*α radiation
μ = 0.09 mm^−1^
*T* = 298 (2) K
0.28 × 0.27 × 0.27 mm

#### Data collection

Bruker SMART APEX area-detector diffractometer
Absorption correction: multi-scan (*SADABS*; Sheldrick, 1996 ▶) *T*_min_ = 0.975, *T*_max_ = 0.976
11531 measured reflections
3094 independent reflections
2579 reflections with *I* > 2σ(*I*)
*R*_int_ = 0.021

#### Refinement

*R*[*F* ^2^ > 2σ(*F* ^2^)] = 0.044
*wR*(*F* ^2^) = 0.130
*S* = 1.05
3094 reflections
196 parameters
1 restraint
H atoms treated by a mixture of independent and constrained refinement
Δρ_max_ = 0.21 e Å^−3^
Δρ_min_ = −0.29 e Å^−3^

### 

Data collection: *SMART* (Siemens, 1996 ▶); cell refinement: *SAINT* (Siemens, 1996 ▶); data reduction: *SAINT*; program(s) used to solve structure: *SHELXS97* (Sheldrick, 2008 ▶); program(s) used to refine structure: *SHELXL97* (Sheldrick, 2008 ▶); molecular graphics: *SHELXTL* (Sheldrick, 2008 ▶); software used to prepare material for publication: *SHELXL97*.

## Supplementary Material

Crystal structure: contains datablocks global, I. DOI: 10.1107/S160053680800130X/su2040sup1.cif

Structure factors: contains datablocks I. DOI: 10.1107/S160053680800130X/su2040Isup2.hkl

Additional supplementary materials:  crystallographic information; 3D view; checkCIF report

## Figures and Tables

**Table 1:** Hydrogen-bond geometry (Å, °)

*D*—H⋯*A*	*D*—H	H⋯*A*	*D*⋯*A*	*D*—H⋯*A*
O2—H2⋯O1^i^	0.82	2.18	2.8470 (14)	138
O2—H2⋯N2^i^	0.82	2.36	3.1008 (16)	150
N3—H3*A*⋯O1^ii^	0.895 (9)	2.561 (11)	3.4172 (16)	160.4 (16)

Symmetry codes: (i) 
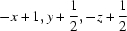
; (ii) 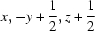.

**Fig. 1.**
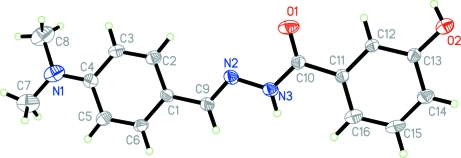
The molecular structure of the title compound, showing the atom labelling scheme and displacement ellipsoids drawn at the 30% probability level.

**Fig. 2.**
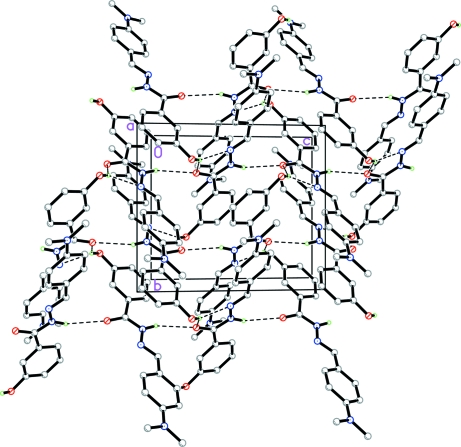
Crystal packing of the title compound view along the a axis [hydrogen bonds are drawn as dotted lines].

## supplementary crystallographic information

### Comment

Schiff base compounds have been widely investigated due to their easy synthesis, versatile structures and wide applications (Sigman & Jacobsen, 1998; Akitsu & Einaga, 2006; Pradeep, 2005; Butcher *et al.*, 2005). The excellent antibacterial and antitumor properties of such compounds have attracted much interest in recent years (Hodnett & Mooney, 1970; Bahner *et al.*, 1968; Merchant & Chothia, 1970). In order to investigate further the structures of such compounds, the new title Schiff base compound is reported on here.

The dihedral angle between the two benzene rings in the molecule (Fig. 1) of the title compound is 9.2 (2)°. In the crystal structure, molecules are linked through intermolecular O–H···O, O–H···N and N–H···O hydrogen bonds (Table 1), forming layers parallel to the *bc* plane (Fig. 2).

### Experimental

The title compound was obtained by stirring of 4-dimethylaminobenzaldehyde (0.1 mmol, 14.9 mg) and 3-hydroxybenzoic acid hydrazide (0.1 mmol, 15.2 mg) in a methanol solution (10 ml) at room temperature. Yellow block-shaped single crystals suitable for X-ray diffraction were formed from the solution after seven days.

### Refinement

H3A was located from a difference Fourier map and refined with the N–H distance restrained to 0.90 (1) Å, and *U*_iso_(H) = 0.08 Å^2^. Other H atoms were positioned geometrically (C–H = 0.93–0.96Å and O–H = 0.82 Å) and treated as riding atoms, with *U*_iso_(H) = 1.2*U*_eq_(C) and 1.5*U*_eq_(O and methyl-C).

### Crystal data

**Table:**

C_16_H_17_N_3_O_2_	*F*_000_ = 600
*M**_r_* = 283.33	*D*_x_ = 1.329 Mg m^−^^3^
Monoclinic, *P*2_1_/*c*	Mo *K*α radiation λ = 0.71073 Å
Hall symbol: -P 2ybc	Cell parameters from 4593 reflections
*a* = 13.397 (3) Å	θ = 2.5–27.7º
*b* = 9.663 (2) Å	µ = 0.09 mm^−^^1^
*c* = 11.183 (2) Å	*T* = 298 (2) K
β = 101.97 (3)º	Block, yellow
*V* = 1416.2 (5) Å^3^	0.28 × 0.27 × 0.27 mm
*Z* = 4	

### Data collection

**Table:**

Bruker SMART APEX area-detector diffractometer	3094 independent reflections
Radiation source: fine-focus sealed tube	2579 reflections with *I* > 2σ(*I*)
Monochromator: graphite	*R*_int_ = 0.021
*T* = 298(2) K	θ_max_ = 27.0º
ω scans	θ_min_ = 2.6º
Absorption correction: multi-scan(SADABS; Sheldrick, 1996)	*h* = −16→17
*T*_min_ = 0.975, *T*_max_ = 0.976	*k* = −12→12
11531 measured reflections	*l* = −14→14

### Refinement

**Table:**

Refinement on *F*^2^	Secondary atom site location: difference Fourier map
Least-squares matrix: full	Hydrogen site location: inferred from neighbouring sites
*R*[*F*^2^ > 2σ(*F*^2^)] = 0.044	H atoms treated by a mixture of independent and constrained refinement
*wR*(*F*^2^) = 0.130	*w* = 1/[σ^2^(*F*_o_^2^) + (0.0773*P*)^2^ + 0.1729*P*] where *P* = (*F*_o_^2^ + 2*F*_c_^2^)/3
*S* = 1.05	(Δ/σ)_max_ < 0.001
3094 reflections	Δρ_max_ = 0.21 e Å^−^^3^
196 parameters	Δρ_min_ = −0.29 e Å^−^^3^
1 restraint	Extinction correction: none
Primary atom site location: structure-invariant direct methods	

### Special details

**Table:**

Geometry. All e.s.d.'s (except the e.s.d. in the dihedral angle between two l.s. planes) are estimated using the full covariance matrix. The cell e.s.d.'s are taken into account individually in the estimation of e.s.d.'s in distances, angles and torsion angles; correlations between e.s.d.'s in cell parameters are only used when they are defined by crystal symmetry. An approximate (isotropic) treatment of cell e.s.d.'s is used for estimating e.s.d.'s involving l.s. planes.
Refinement. Refinement of *F*^2^ against ALL reflections. The weighted *R*-factor *wR* and goodness of fit *S* are based on *F*^2^, conventional *R*-factors *R* are based on *F*, with *F* set to zero for negative *F*^2^. The threshold expression of *F*^2^ > σ(*F*^2^) is used only for calculating *R*-factors(gt) *etc*. and is not relevant to the choice of reflections for refinement. *R*-factors based on *F*^2^ are statistically about twice as large as those based on *F*, and *R*- factors based on ALL data will be even larger.

### Fractional atomic coordinates and isotropic or equivalent isotropic displacement parameters (Å^2^)

**Table:**

	*x*	*y*	*z*	*U*_iso_*/*U*_eq_	
O1	0.51231 (8)	0.27675 (11)	0.29293 (9)	0.0574 (3)	
O2	0.28657 (7)	0.69755 (10)	0.22473 (9)	0.0486 (3)	
H2	0.3315	0.7006	0.1849	0.073*	
N1	0.97117 (10)	−0.30631 (15)	0.63913 (13)	0.0654 (4)	
N2	0.60441 (8)	0.13189 (10)	0.48763 (10)	0.0416 (3)	
N3	0.52359 (8)	0.22033 (11)	0.49046 (10)	0.0420 (3)	
C1	0.72192 (9)	−0.03254 (12)	0.59700 (11)	0.0379 (3)	
C2	0.78863 (10)	−0.03161 (13)	0.51647 (11)	0.0430 (3)	
H2A	0.7774	0.0302	0.4513	0.052*	
C3	0.87041 (10)	−0.11981 (14)	0.53134 (12)	0.0445 (3)	
H3	0.9138	−0.1157	0.4764	0.053*	
C4	0.89036 (9)	−0.21613 (13)	0.62715 (12)	0.0425 (3)	
C5	0.82405 (10)	−0.21577 (14)	0.70911 (12)	0.0456 (3)	
H5	0.8352	−0.2770	0.7747	0.055*	
C6	0.74284 (10)	−0.12595 (14)	0.69358 (11)	0.0424 (3)	
H6	0.7004	−0.1277	0.7496	0.051*	
C7	0.99119 (16)	−0.4061 (2)	0.73523 (17)	0.0793 (6)	
H7A	1.0129	−0.3597	0.8121	0.119*	
H7B	1.0439	−0.4679	0.7219	0.119*	
H7C	0.9302	−0.4578	0.7363	0.119*	
C8	1.04154 (15)	−0.2992 (2)	0.5584 (2)	0.0903 (7)	
H8A	1.0063	−0.3194	0.4764	0.135*	
H8B	1.0952	−0.3655	0.5835	0.135*	
H8C	1.0702	−0.2079	0.5612	0.135*	
C9	0.63422 (9)	0.05897 (13)	0.58348 (11)	0.0411 (3)	
H9	0.5988	0.0645	0.6466	0.049*	
C10	0.48449 (9)	0.29458 (12)	0.38962 (12)	0.0397 (3)	
C11	0.40510 (9)	0.39961 (12)	0.40150 (11)	0.0375 (3)	
C12	0.38258 (9)	0.49733 (12)	0.30887 (11)	0.0375 (3)	
H12	0.4168	0.4954	0.2446	0.045*	
C13	0.30939 (9)	0.59770 (12)	0.31174 (11)	0.0380 (3)	
C14	0.25681 (10)	0.59887 (14)	0.40629 (12)	0.0453 (3)	
H14	0.2067	0.6651	0.4080	0.054*	
C15	0.27927 (11)	0.50121 (16)	0.49776 (13)	0.0530 (4)	
H15	0.2440	0.5021	0.5611	0.064*	
C16	0.35350 (10)	0.40181 (14)	0.49689 (12)	0.0469 (3)	
H16	0.3686	0.3372	0.5596	0.056*	
H3A	0.5095 (14)	0.2388 (19)	0.5637 (11)	0.080*	

### Atomic displacement parameters (Å^2^)

**Table:**

	*U*^11^	*U*^22^	*U*^33^	*U*^12^	*U*^13^	*U*^23^
O1	0.0646 (6)	0.0592 (6)	0.0572 (6)	0.0197 (5)	0.0327 (5)	0.0044 (5)
O2	0.0480 (5)	0.0487 (5)	0.0530 (6)	0.0081 (4)	0.0192 (4)	0.0094 (4)
N1	0.0552 (7)	0.0751 (9)	0.0698 (8)	0.0289 (7)	0.0219 (6)	0.0220 (7)
N2	0.0369 (5)	0.0388 (6)	0.0501 (6)	0.0050 (4)	0.0114 (4)	−0.0082 (4)
N3	0.0380 (5)	0.0417 (6)	0.0485 (6)	0.0072 (4)	0.0137 (5)	−0.0065 (5)
C1	0.0368 (6)	0.0382 (6)	0.0391 (6)	−0.0001 (5)	0.0087 (5)	−0.0057 (5)
C2	0.0452 (7)	0.0451 (7)	0.0400 (6)	0.0056 (5)	0.0115 (5)	0.0069 (5)
C3	0.0427 (7)	0.0535 (8)	0.0407 (7)	0.0056 (5)	0.0162 (5)	0.0024 (5)
C4	0.0388 (6)	0.0447 (7)	0.0432 (7)	0.0046 (5)	0.0067 (5)	0.0001 (5)
C5	0.0467 (7)	0.0504 (7)	0.0397 (7)	0.0023 (6)	0.0085 (5)	0.0086 (5)
C6	0.0428 (7)	0.0499 (7)	0.0373 (6)	−0.0026 (5)	0.0149 (5)	−0.0027 (5)
C7	0.0834 (12)	0.0834 (13)	0.0713 (11)	0.0421 (10)	0.0162 (9)	0.0196 (9)
C8	0.0656 (11)	0.1028 (15)	0.1139 (16)	0.0410 (11)	0.0449 (11)	0.0305 (13)
C9	0.0394 (6)	0.0403 (6)	0.0456 (7)	0.0004 (5)	0.0131 (5)	−0.0074 (5)
C10	0.0359 (6)	0.0369 (6)	0.0497 (7)	0.0000 (5)	0.0165 (5)	−0.0046 (5)
C11	0.0332 (6)	0.0371 (6)	0.0442 (7)	−0.0012 (5)	0.0122 (5)	−0.0055 (5)
C12	0.0352 (6)	0.0394 (6)	0.0412 (6)	−0.0024 (5)	0.0156 (5)	−0.0044 (5)
C13	0.0345 (6)	0.0378 (6)	0.0423 (6)	−0.0024 (5)	0.0095 (5)	−0.0018 (5)
C14	0.0393 (6)	0.0472 (7)	0.0530 (7)	0.0094 (5)	0.0182 (5)	−0.0002 (6)
C15	0.0532 (8)	0.0630 (9)	0.0511 (8)	0.0150 (6)	0.0302 (6)	0.0071 (6)
C16	0.0486 (7)	0.0502 (7)	0.0464 (7)	0.0102 (6)	0.0199 (6)	0.0079 (6)

### Geometric parameters (Å, °)

**Table:**

O1—C10	1.2267 (15)	C6—H6	0.9300
O2—C13	1.3593 (15)	C7—H7A	0.9600
O2—H2	0.8200	C7—H7B	0.9600
N1—C4	1.3745 (17)	C7—H7C	0.9600
N1—C7	1.427 (2)	C8—H8A	0.9600
N1—C8	1.436 (2)	C8—H8B	0.9600
N2—C9	1.2757 (16)	C8—H8C	0.9600
N2—N3	1.3848 (14)	C9—H9	0.9300
N3—C10	1.3475 (17)	C10—C11	1.4958 (16)
N3—H3A	0.895 (9)	C11—C16	1.3864 (18)
C1—C6	1.3907 (17)	C11—C12	1.3878 (17)
C1—C2	1.3942 (17)	C12—C13	1.3842 (17)
C1—C9	1.4531 (17)	C12—H12	0.9300
C2—C3	1.3706 (17)	C13—C14	1.3868 (17)
C2—H2A	0.9300	C14—C15	1.3783 (19)
C3—C4	1.4025 (18)	C14—H14	0.9300
C3—H3	0.9300	C15—C16	1.3841 (18)
C4—C5	1.4025 (19)	C15—H15	0.9300
C5—C6	1.3744 (18)	C16—H16	0.9300
C5—H5	0.9300		
			
C13—O2—H2	109.5	H7B—C7—H7C	109.5
C4—N1—C7	121.53 (13)	N1—C8—H8A	109.5
C4—N1—C8	120.99 (13)	N1—C8—H8B	109.5
C7—N1—C8	117.43 (13)	H8A—C8—H8B	109.5
C9—N2—N3	115.57 (10)	N1—C8—H8C	109.5
C10—N3—N2	118.66 (10)	H8A—C8—H8C	109.5
C10—N3—H3A	122.7 (12)	H8B—C8—H8C	109.5
N2—N3—H3A	117.2 (12)	N2—C9—C1	121.94 (11)
C6—C1—C2	116.93 (11)	N2—C9—H9	119.0
C6—C1—C9	120.27 (11)	C1—C9—H9	119.0
C2—C1—C9	122.80 (11)	O1—C10—N3	121.76 (11)
C3—C2—C1	121.39 (12)	O1—C10—C11	121.68 (12)
C3—C2—H2A	119.3	N3—C10—C11	116.56 (10)
C1—C2—H2A	119.3	C16—C11—C12	119.87 (11)
C2—C3—C4	121.75 (11)	C16—C11—C10	123.77 (11)
C2—C3—H3	119.1	C12—C11—C10	116.35 (10)
C4—C3—H3	119.1	C13—C12—C11	120.32 (10)
N1—C4—C5	122.05 (12)	C13—C12—H12	119.8
N1—C4—C3	121.11 (12)	C11—C12—H12	119.8
C5—C4—C3	116.84 (11)	O2—C13—C12	122.41 (10)
C6—C5—C4	120.73 (12)	O2—C13—C14	117.75 (11)
C6—C5—H5	119.6	C12—C13—C14	119.84 (11)
C4—C5—H5	119.6	C15—C14—C13	119.55 (11)
C5—C6—C1	122.33 (11)	C15—C14—H14	120.2
C5—C6—H6	118.8	C13—C14—H14	120.2
C1—C6—H6	118.8	C14—C15—C16	121.09 (12)
N1—C7—H7A	109.5	C14—C15—H15	119.5
N1—C7—H7B	109.5	C16—C15—H15	119.5
H7A—C7—H7B	109.5	C15—C16—C11	119.32 (12)
N1—C7—H7C	109.5	C15—C16—H16	120.3
H7A—C7—H7C	109.5	C11—C16—H16	120.3

### Hydrogen-bond geometry (Å, °)

**Table:**

*D*—H···*A*	*D*—H	H···*A*	*D*···*A*	*D*—H···*A*
O2—H2···O1^i^	0.82	2.18	2.8470 (14)	138
O2—H2···N2^i^	0.82	2.36	3.1008 (16)	150
N3—H3A···O1^ii^	0.895 (9)	2.561 (11)	3.4172 (16)	160.4 (16)

Symmetry codes: (i) −*x*+1, *y*+1/2, −*z*+1/2; (ii) *x*, −*y*+1/2, *z*+1/2.
